# Healthcare-Associated Infections (HAIs) in the Elderly: Molecular Mechanisms of Immunosenescence and Clinical, Nutritional and Therapeutic Implications

**DOI:** 10.3390/ijms26199649

**Published:** 2025-10-03

**Authors:** Livia Moffa, Claudio Tana

**Affiliations:** 1 Infectious Disease Clinic, University Hospital of Chieti and ASL2 Lanciano Vasto Chieti, 66100 Chieti, CH, Italy; 2 Internal Medicine Unit, Eastern Hospital, ASL Taranto, 74024 Manduria, TA, Italy

**Keywords:** immunosenescence, inflammaging, microbiota, epigenesis, genetic, cross infection, malnutrition

## Abstract

Healthcare-associated infections (HAIs) in the elderly represent a growing clinical and public health concern, primarily driven by age-related biological remodeling. Key mechanisms include immunosenescence, inflammaging, gut microbiota dysbiosis, and profound metabolic and epigenetic alterations, all of which progressively weaken host defense and resilience to pathogens. In this review, we delineate the molecular pathways underlying these processes, with particular attention to impaired innate and adaptive immune responses, dysfunctional cellular signaling, and disrupted immunometabolic networks that increase susceptibility to multidrug-resistant organisms and aggravate clinical outcomes in older patients. We also address the synergistic impact of frailty-related factors such as malnutrition, multimorbidity, and polypharmacy on infection risk. Finally, we discuss emerging translational perspectives, including nutritional interventions and microbiota-targeted strategies aimed at restoring immune competence and reducing infection burden. By integrating molecular mechanisms with clinical implications, this review highlights innovative opportunities for personalized prevention and management of HAIs in the aging population.

## 1. Introduction

Healthcare-associated infections (HAIs) in the elderly exhibit a disproportionately higher incidence and severity compared to younger populations, representing a major clinical and economic burden. HAIs affect ~4.6–9.3% of hospitalized patients in Europe and ~5–15% in high-income countries (up to 10% in emerging economies), with particularly high burdens in ICUs (up to 37% of inpatients), and an estimated impact in the United States of ~1.7 million cases and ~98,000 deaths per year [1]. It has been shown that the risk of HAIs rises progressively with age. In particular, prevalence reaches 11.5% among patients over 85 years, compared with 7.4% in those younger than 65 years [2]. Common pathogens include *S. aureus* (including Methicillin-Resistant Staphylococcus aureus, MRSA), *Enterobacterales* (Extended-Spectrum Beta-Lactamase, ESBL/carbapenemase producers), *P. aeruginosa*, and *Acinetobacter* spp., with increasingly resistant profiles [1]. These infections are associated with increased mortality, prolonged hospitalization, and frequent recurrences due to multidrug-resistant organisms (MDROs) [1]. The underlying determinants extend beyond exposure to healthcare environments, encompassing immune system remodeling, notably immunosenescence, impaired regulatory cell function, and progressive loss of clonal diversity. Collectively, these mechanisms can predispose older adults to poorer outcomes and escalating healthcare costs [2].

Frailty and multimorbidity act as accelerators of immune aging, exacerbating vulnerability to HAIs by amplifying molecular pathways of immunosenescence. Key drivers include mitochondrial dysfunction, persistence of the senescence-associated secretory phenotype (SASP), chronic low-grade inflammation (*inflammaging*), and aberrant activation of innate immune sensors such as the cGAS–STING pathway and the NLRP3 inflammasome [3]. These molecular derangements compromise host defense, reduce microbial clearance, and promote an environment permissive to persistent infection [3]. Beyond intrinsic immune defects, nutritional and functional impairments play a pivotal role. Malnutrition and sarcopenia blunt phagocyte function, while dysphagia, immobility, and the frequent use of indwelling medical devices favor micro-aspiration, biofilm formation, and colonization by MDROs [4].

This narrative review provides a comprehensive analysis of the molecular mechanisms linking aging and HAIs, with a particular focus on immunosenescence, inflammaging, metabolic and epigenetic alterations, and microbiota dysbiosis. In parallel, we address the clinical, nutritional, and therapeutic implications, highlighting potential preventive and restorative strategies from immunonutrition and microbiota-targeted interventions to innovative immunomodulatory approaches. By bridging mechanistic insights with clinical translation, we aim to delineate future directions for personalized prevention, diagnosis, and management of HAIs in the geriatric population.

## 2. Methods

We conducted a literature search in PubMed/MEDLINE and Scopus using combinations of the following keywords: “healthcare-associated infections,” “elderly,” “older adults,” “immunosenescence,” “inflammaging,” “frailty,” “multimorbidity,” “polypharmacy,” “epigenetics,” “nutrition,” “malnutrition,” “probiotics,” “therapeutic implications,” and “preventive strategies”.

Articles were selected if they addressed molecular mechanisms, clinical aspects, or preventive and therapeutic strategies of healthcare-associated infections in older adults. Publications not in English, conference abstracts, and non-peer-reviewed sources were excluded. The reference lists of retrieved articles were also screened to identify additional relevant studies.

## 3. Immunosenescence and Innate Immunity Dysfunction

### 3.1. Altered Toll-like Receptor (TLR) Signaling and Antigen Presentation

The first level of impairment concerns innate immune circuits, which constitute the initial barrier to HAIs [5]. With advancing age, the innate immune system undergoes profound remodeling, resulting in a paradoxical state of hyporesponsiveness to pathogens combined with chronic low-grade hyperinflammation [5]. This is reflected in attenuated Toll-like receptor (TLR) signaling and less efficient antigen presentation by dendritic cells (DCs) and monocytes [6]. Specifically, aged DCs and monocytes display reduced TLR-dependent reactivity, and stimulation of TLR7 and TLR9 results in diminished production of interferon-α (IFN-α) and interleukin-12 (IL-12), impairing antiviral and antibacterial responses as well as effective antibody production [7]. In parallel, antigen presentation capacity is compromised by reduced expression of major histocompatibility complex class II (MHC-II) and co-stimulatory molecules (CD80/CD86), along with attenuated cytokine secretion [8]. This leads to inefficient priming of cytotoxic T lymphocytes (CTLs) and T helper cells, thereby weakening adaptive immune responses [9].

### 3.2. Impaired Neutrophil Chemotaxis and Phagocytosis, Decline in Natural Killer (Nk) Cell Cytotoxicity and Dysregulated Complement Activation

Defective pathogen sensing and antigen presentation limit early immune activation, setting the stage for broader innate dysfunctions [10]. Neutrophils, which represent the first line of defense against bacterial and fungal pathogens, display impaired chemotaxis, reduced phagocytic capacity, and an attenuated oxidative burst, ultimately limiting their ability to contain infections and contributing to inappropriate tissue damage [10]. Similarly, natural killer (NK) cells undergo functional decline, with a progressive reduction in their cytotoxic activity and cytokine secretion, resulting in decreased clearance of virally infected and malignant cells [11]. In addition, the complement system becomes increasingly dysregulated, with excessive or misdirected activation that promotes the release of pro-inflammatory anaphylatoxins (C3a, C5a) and membrane attack complexes, thereby amplifying bystander inflammation and collateral tissue damage [12].

Collectively, these alterations contribute to a paradoxical state of immune senescence characterized by reduced antimicrobial defense on the one hand and chronic, low-grade inflammatory activity on the other. The net effect is delayed pathogen recognition, suboptimal microbial clearance, and excessive tissue damage, all of which contribute to the severity and persistence of HAIs, particularly in the presence of invasive devices [13]. These innate deficits set the stage for further impairment at the adaptive level [12].

## 4. Adaptive Immune Remodeling

### 4.1. T-Cell Exhaustion, Reduced Naïve T-Cell Pool, and Clonal Senescence

Adaptive immunity is composed of T and B lymphocytes, responsible for cellular and humoral responses, respectively. With advancing age, this compartment undergoes profound modifications that compromise the efficiency of immune responses and increase the susceptibility of older adults to HAIs [14,15].

A key aspect of immunosenescence involves thymic involution. With aging, both the cortex and medulla of the thymus are progressively replaced by adipose tissue, leading to a drastic reduction in the production of naïve T lymphocytes. This reduction, which becomes evident as early as 40–54 years of age and is further accentuated in the 55–69- and 70–90 years age groups, limits the number of new T cells entering circulation and thus narrows the ability to respond to novel antigens [16].

### 4.2. Imbalance Between Th1/Th2/Th17 and Regulatory T Cells

In parallel, a decline is observed in the total number of T lymphocytes, their clonal diversity, and the intensity of intracellular signaling. The reduced diversity of the T cell receptor repertoire becomes particularly marked after the age of 65, significantly impairing the ability to recognize emerging pathogens. Phenotypically, aging is associated with a decrease in CD4+ T lymphocytes, an increase in CD8+ cells, and a progressive loss of CD28 expression, a crucial costimulatory molecule. This imbalance compromises IL-2 production and reduces the efficiency of T helper–B cell cooperation, resulting in diminished antibody production [17].

Immune homeostasis is further altered by the behavior of regulatory T cells (Tregs). Although their numbers increase with age, their functionality is reduced, resulting in a state of dysregulation that limits effective responses against new antigens while simultaneously favoring chronic inflammation [18]. The balance among T helper subsets is also profoundly affected: Th1 responses, critical for controlling intracellular pathogens, are weakened, whereas Th2 and Th17 responses tend to prevail, fueling a pro-inflammatory and dysfunctional state [19].

### 4.3. B-Cell Repertoire Contraction, Reduced Antibody Affinity, Defective Germinal Center Reactions and Impaired Memory Responses

Imbalanced helper subsets and dysfunctional Treg activity exacerbate inflammation and reduce coordination with B cells [20]. In older adults, the clonal repertoire narrows and the ability to produce high-affinity antibodies is significantly reduced. Although total immunoglobulin levels may appear increased, the proportion of antigen-specific antibodies generated following antigen exposure is markedly lower [20].

Another critical mechanism involves impaired cooperation between follicular helper T cells (Tfh) and B cells, which is indispensable for germinal center organization and function. With aging, germinal center activity in lymph nodes and spleen becomes less efficient, resulting in reduced generation of long-lived plasma cells and memory B cells [21]. This deficit translates into diminished effectiveness of secondary responses, both against reinfections and in response to vaccination [22].

Moreover, age-related changes affect the expression of activation-induced cytidine deaminase (AID) and other enzymes crucial for class-switch recombination and somatic hypermutation. As a result, antibody affinity maturation is compromised, leading to a predominance of low-affinity IgM and reduced isotype switching toward IgG and IgA, both essential for mucosal and systemic defense [23]. These alterations not only weaken protection against novel pathogens but also reduce the effectiveness of vaccines specifically designed for older adults.

Recent studies further highlight an age-dependent expansion of so-called “age-associated B cells” (ABCs), a subset characterized by expression of CD11c and T-bet, which exhibit pro-inflammatory properties and reduced capacity to generate protective responses [24]. The accumulation of ABCs contributes to systemic inflammation and autoantibody production, adding another layer of dysregulation to the humoral immune system in older individuals.

In summary, the remodeling of the B-cell compartment during aging involves repertoire contraction, defective germinal center responses, impaired antibody maturation, and the emergence of dysfunctional subsets. Together with T-cell alterations, these changes profoundly limit both antigen recognition and the ability to mount durable and protective immune responses. Collectively, these mechanisms play a decisive role in the increased incidence and severity of HAIs in the elderly population [20,21,22,23].

Imbalanced helper subsets and dysfunctional Treg activity exacerbate inflammation and reduce coordination with B cells [20].

## 5. Inflammaging and Chronic Low-Grade Inflammation

### 5.1. NF-κB Hyperactivation and Persistent Cytokine Production

Subclinical inflammation emerges as a transversal amplifier of immunosenescence-related vulnerability [25]. “Inflammaging” represents a state of chronic, low-grade inflammation that progressively develops with advancing age, and is considered a hallmark of immune aging [25]. This condition significantly contributes to increasing the vulnerability of older adults to HAIs.

Several molecular mechanisms underlie this process. Among them, a central role is played by the persistent activation of the transcription factor NF-κB, a key regulator of inflammatory responses. In older individuals, its chronic activation results in sustained elevation of pro-inflammatory cytokines such as IL-6, TNF-α, and IL-1β, even in the absence of infectious stimuli [25].

### 5.2. Senescence-Associated Secretory Phenotype (SASP)

With aging, there is also a progressive accumulation of senescent cells that acquire a SASP. This phenotype is characterized by the release of cytokines, chemokines, metalloproteinases, and growth factors that sustain the chronic inflammatory state. Although SASP initially exerts protective functions by promoting tissue repair and the clearance of damaged cells, its long-term persistence promotes immunosenescence and alters tissue microenvironments, favoring dysfunctional conditions [26].

### 5.3. Role of Mitochondrial Dysfunction, ROS and Systemic Effects on Infection Risk and Tissue Repair

Another relevant mechanism involves the progressive impairment of mitochondrial function. With age, a decline in respiratory efficiency is observed, accompanied by increased production of reactive oxygen species (ROS). Excessive ROS cause oxidative damage to DNA, proteins, and lipids, while simultaneously amplifying inflammatory responses through activation of NF-κB and the NLRP3 inflammasome [27]. Activation of the NLRP3 inflammasome promotes the maturation and release of IL-1β and IL-18, cytokines that perpetuate chronic low-grade inflammation. This pathway directly links mitochondrial dysfunction to inflammaging, creating a self-sustaining cycle of oxidative stress and immune dysregulation. In the context of HAIs, mitochondrial dysfunction and inflammaging converge to increase susceptibility by weakening antimicrobial defenses and impairing tissue repair. Chronic low-grade inflammation compromises the ability to mount effective immune responses against pathogenic microorganisms, thereby raising the risk of nosocomial infections. At the same time, the persistent inflammatory milieu delays wound healing and postoperative recovery, ultimately heightening the likelihood of infectious complications and overall clinical vulnerability in older hospitalized patients [23,27].

In summary, inflammaging represents a key component of immunosenescence: a persistent, subclinical inflammation that not only favors the onset and severity of HAIs but also compromises the reparative and regenerative capacity of the organism in older individuals [23].

## 6. Metabolic Rewiring and Immunometabolism

### 6.1. mTOR and AMPK Signaling Imbalance

Building on this chronic inflammatory background, age-related metabolic alterations further shape immune cell function, linking inflammaging to profound changes in immunometabolism, which focuses on the metabolic pathways that regulate immune cell activation, differentiation, and effector functions, and represents a fundamental determinant of host defense [28]. Aging profoundly alters these mechanisms, leading to a “metabolic rewiring” that, in close interaction with immunosenescence, contributes to the increased susceptibility of older adults to HAIs.

In elderly individuals, persistent hyperactivation of the mechanistic target of rapamycin (mTOR) pathway has been documented, together with reduced activation of AMP-activated protein kinase (AMPK). The mTOR pathway promotes anabolic processes and cellular growth, whereas AMPK supports catabolic pathways and adaptation to stress [28,29]. The imbalance in favor of mTOR results in chronic inflammation and reduced immune cell responsiveness to infectious stimuli, typical of hospital settings. While these pathways are increasingly recognized as targets for immunosenescence, their clinical translation is still preliminary. Most available data derive from animal models or indirect evidence from metabolic diseases, and large-scale trials in elderly inpatients are still lacking.

### 6.2. Defective Autophagy, Mitophagy in Immune Cells and Altered Bioenergetics of T and B Lymphocytes

Autophagy and mitophagy that are essential for the clearance of damaged cellular components and for the maintenance of cellular homeostasis, are also impaired with aging [30,31]. Their inefficiency leads to the accumulation of dysfunctional mitochondria and increased production of ROS, which amplify oxidative stress, impair antigen presentation, and reduce the capacity of immune cells to eliminate intracellular microorganisms.

Metabolic rewiring also affects adaptive immunity. In naïve T cells, the ability to switch from oxidative metabolism to aerobic glycolysis during clonal expansion is impaired, leading to slower and less effective responses against hospital-acquired pathogens. In B lymphocytes, bioenergetic alterations hinder differentiation into plasma cells and the production of high-affinity antibodies, thereby reducing vaccine efficacy and protection against recurrent infections in hospitalized older adults. These changes not only impair antimicrobial responses but also alter tolerance mechanisms, promoting autoimmunity and chronic low-grade inflammation. Both aspects converge to increase the frequency, severity, and complexity of HAIs in this population [32].

The metabolic rewiring of immune cells in the elderly therefore represents not only a biological phenomenon but also a clinical determinant of immune frailty, and could explain the greater severity and higher risk of complications associated with HAIs in older adults.

## 7. Epigenetic Alterations and Immune Aging

### 7.1. DNA Methylation Drift, Immunosenescence Biomarkers and Histone Modifications Affecting Inflammatory Gene Expression

The aging of the immune system is not solely determined by genetic factors but also by epigenetic modifications, namely changes in gene expression that occur without altering the DNA sequence. Among the key epigenetic determinants are lifestyle and environmental factors. These changes, which progressively accumulate over the course of life, substantially contribute to immunosenescence and to the increased susceptibility of older adults to HAIs [33]. It has been increasingly recognized that “successful” immune aging appears to rely less on strictly genetic determinants and more on the capacity to generate effective immune responses against pathogens, thereby mitigating cellular damage and tissue inflammation [34].

In this context, DNA methylation (DNAm) represents one of the most extensively studied epigenetic mechanisms. Methyl groups can be added to or removed from DNA, thereby regulating gene expression. With advancing age, this process undergoes a “drift” characterized by global hypomethylation and site-specific hypermethylation. Such alterations influence the expression of genes involved in lymphocyte proliferation and functionality, ultimately impairing immune responses. Notably, DNA methylation has been proposed as a biomarker of immunosenescence and of susceptibility to HAIs [35].

In addition to DNA, histones undergo a wide range of post-translational modifications, most notably acetylation and methylation, catalyzed by enzymes such as histone acetyltransferases (HATs), histone deacetylases (HDACs), and histone methyltransferases (DNMT). In older individuals, these epigenetic alterations promote the sustained activation of NF-κB–mediated inflammatory pathways, a defining feature of the “inflammaging” phenotype. This chronic pro-inflammatory state not only heightens susceptibility to hospital-acquired infections but also compromises tissue repair and regenerative capacity [36].

### 7.2. Non-Coding RNAs (miRNAs, lncRNAs) in Immune Regulation and Epigenetic Control of Antimicrobial Responses

An additional layer of epigenetic regulation is represented by non-coding RNAs, particularly microRNAs (miRNAs) and long non-coding RNAs (lncRNAs). miRNAs, which play a pivotal role in regulating both innate and adaptive immune responses, display altered expression profiles in the elderly [37]. Their dysregulation impairs T-cell activation and diminishes the organism’s ability to mount effective defenses against novel infections. Similarly, specific lncRNAs modulate the transcription of genes essential for immune homeostasis and, when altered, contribute to the establishment of a chronic inflammatory state [37]. Epigenetic alterations also directly affect the efficiency of antimicrobial responses. DNA methylation and histone modifications regulate, for example, the expression of pathogen recognition receptors (PRRs), antimicrobial molecules, and mediators of innate immunity. With advancing age, the dysregulation of these mechanisms contributes to a diminished capacity to eliminate pathogens and to increased severity of nosocomial infections [38].

In summary, epigenetic modifications represent a crucial component of the immunosenescence process. By modulating inflammatory and antimicrobial circuits, and interacting with other mechanisms of immune aging, they help explain the high incidence and severity of HAIs in the elderly population [39].

## 8. Gut Microbiota Dysbiosis and the Gut–Immune Axis

### 8.1. Age-Related Shifts in Microbial Composition and Diversity

Age-related dysbiosis integrates with immunosenescence to amplify infection risk. Aging is associated with marked alterations in microbial ecology, including reduced alpha diversity, loss of key short-chain fatty acid (SCFA) producers such as *Faecalibacterium prausnitzii* and *Roseburia* spp., and an overgrowth of opportunistic *Enterobacterales* [40]. These compositional shifts impair epithelial barrier integrity, decrease local SCFA availability, and promote a pro-inflammatory milieu that predisposes to pathogen overgrowth and translocation. Hospital-related factors including dietary restrictions, polypharmacy, multimorbidity, and cumulative exposure to broad-spectrum antibiotics, further exacerbate this dysbiotic state. The result is a permissive niche for colonization by *Clostridioides difficile*, ESBL-producing Enterobacteriaceae, carbapenem-resistant *Klebsiella pneumoniae*, and vancomycin-resistant *Enterococcus faecium* (VRE), which significantly increases the incidence and severity of HAIs in the elderly [41,42]. In institutionalized elderly patients, reduced microbial diversity has been associated with a higher incidence of bacteremia and an increased risk of *Clostridioides difficile* infections, particularly following hospital-based antibiotic therapies. [43].

### 8.2. Reduced Short-Chain Fatty Acids and Immune Regulation

One of the most critical consequences of age-related gut microbiota alterations is the reduced production of SCFAs, particularly butyrate, acetate, and propionate. These metabolites are essential not only for maintaining epithelial barrier integrity but also for immune regulation through mechanisms such as activation of G-protein–coupled receptors (e.g., GPR41, GPR43) and inhibition of HDACs. These pathways promote the differentiation and function of Tregs and support mucosal immune tolerance. SCFA deficiency therefore disrupts epithelial homeostasis, impairs Treg-mediated control of inflammation, and amplifies pro-inflammatory responses, creating a permissive environment for hospital-acquired pathogens and predisposing to recurrent *Clostridioides difficile* infection [44]. Several studies have also highlighted that SCFA-deficient microbial profiles are associated with decolonization failures and a high likelihood of reinfections, thereby complicating the clinical course of hospitalized older adults [45].

### 8.3. Increased Gut Permeability and Endotoxin Translocation

Concomitantly, aging induces significant structural alterations of the intestinal mucosa. Impairment of tight junctions, reduced mucus secretion, and defective epithelial regeneration collectively contribute to the “leaky gut” phenotype, characterized by increased intestinal barrier permeability. This condition facilitates the translocation of lipopolysaccharides (LPS) and other pathogen-associated molecular patterns (PAMPs), driving chronic endotoxemia and the activation of inflammatory pathways such as NF-κB and NLRP3 [46]. Both clinical and experimental evidence confirm that these mechanisms are strongly associated with an increased incidence of enteric-derived bacteremia, episodes of sepsis, and a more severe clinical course, particularly in the context of polypharmacy and pronounced dysbiosis [46].

### 8.4. Interplay Between Dysbiosis, Inflammaging, and HAIs

Intestinal dysbiosis further amplifies the phenomenon of inflammaging, sustaining a chronically inflamed yet paradoxically inefficient mucosal microenvironment that fails to control pathogens effectively [47]. Continuous input of LPS together with the deficiency of metabolites such as SCFAs promotes persistent colonization by multidrug-resistant microorganisms, including VRE, ESBL-producing *Enterobacteriaceae*, and carbapenem-resistant *Enterobacteriaceae* (CRE), leading to a marked increase in the incidence and severity of HAIs [48]. Within this context, prolonged hospitalization acts as an additional amplifier, closing a vicious cycle that renders older adults particularly vulnerable to HAIs [49]. Recent “microbiota-directed” strategies are opening new therapeutic perspectives. Fecal microbiota transplantation (FMT), already implemented successfully in several specialized centers, has been shown to significantly reduce *Clostridioides difficile* recurrences and to partially restore colonization resistance against MDR pathogens, outlining a promising preventive and therapeutic approach for HAIs [50]. In summary, the progressive dysbiosis that accompanies aging represents a key determinant of immune frailty in older adults. The combination of reduced microbial diversity, SCFA deficiency, impaired intestinal barrier function, and enhanced inflammaging convincingly explains the increased susceptibility to HAIs, the higher frequency of persistent colonization, and the greater severity of nosocomial infections observed in the geriatric population.

The key molecular mechanisms of immunosenescence, their functional consequences, and their impact on HAIs in older adults are detailed in Table 1.

## 9. Clinical and Nutritional Implications

### 9.1. Malnutrition, Sarcopenia, and Immune Vulnerability

Translating molecular mechanisms into clinical contexts highlights modifiable determinants of infection risk. Adequate nutrition is a fundamental requirement for maintaining full immune competence in older adults and represents a cornerstone in the prevention and control of nosocomial infections [51]. Deficiencies in essential nutrients, particularly protein-energy malnutrition, together with sarcopenia, comorbidities, microbiota alteration and polypharmacy, collectively impair the integrity of host defense barriers and facilitate colonization by MDROs [52]. This combination of factors significantly increases the risk of HAIs and worsens clinical outcomes.

Protein-energy malnutrition is highly prevalent among hospitalized older adults, with reported rates ranging from 12% to 50% [53]. This impairment leads to a reduction in protein synthesis, which is indispensable for the production of cytokines and antibodies, thereby compromising both innate and adaptive immune responses [54]. As a result, the immune system undergoes progressive weakening, with impaired T- and B-cell function and consequent compromise of both innate and adaptive immunity, and predisposes older adults to heightened vulnerability to HAIs [23].

A direct consequence of malnutrition is often sarcopenia, defined as the progressive loss of muscle mass, strength, and function. This condition, which is highly common in the geriatric population, leads to a reduced availability of essential amino acids necessary to sustain the inflammatory response and gluconeogenesis [55].

Several observational studies and meta-analyses have demonstrated that both malnutrition and sarcopenia are independent risk factors for sepsis and nosocomial infections, particularly pneumonia, urinary tract infections, surgical site infections, and stroke-associated infections (SAI). For instance, in a large cohort of older patients with acute ischemic stroke, pre-stroke sarcopenia risk was independently associated with a significantly higher incidence of pneumonia and urinary tract infections during hospitalization [56]. In another study of elderly patients hospitalized with community-acquired pneumonia, malnutrition was linked not only to longer hospital stays and higher readmission rates, but also increased in-hospital mortality [57]. Collectively, these findings underscore malnutrition and sarcopenia as independent contributors to immune frailty and adverse outcomes in geriatric inpatients.

### 9.2. Polypharmacy and Its Impact on Microbiota and Immunity

Polypharmacy, commonly defined as the concomitant use of five or more medications, is a hallmark of geriatric care and exerts multifaceted effects on host–microbiota–immune interactions [58]. Several pharmacological classes frequently prescribed in older adults including antibiotics, proton pump inhibitors, nonsteroidal anti-inflammatory drugs, psychotropics, and antidiabetics have been demonstrated to profoundly alter intestinal microbial diversity and metabolic capacity [58]. These drug-induced perturbations reduce the abundance of commensal, SCFA-producing taxa while facilitating the expansion of pathobionts, thereby impairing epithelial barrier integrity and promoting the systemic translocation of pathogen-associated molecular patterns such as lipopolysaccharides [59]. This dysbiotic milieu can contribute to “inflammaging” and accelerate immunosenescence. Some evidence link also chronic exposure to multiple pharmacological agents to a reduced vaccine immunogenicity [60].

Among specific examples, proton pump inhibitors have been shown to reproducibly alter gut microbial ecology and to predispose to Clostridioides difficile infection, further exemplifying the translational consequences of polypharmacy-induced dysbiosis [61].

Although direct studies linking polypharmacy with nosocomial infection rates in elderly inpatients are still limited, some observational studies show that polypharmacy is associated with higher incidence of pneumonia-related hospitalizations and worse clinical utilization metrics [62] and that hospitalised geriatric populations with high medication burdens present with more severe comorbidities and worse outcomes overall [63].

Collectively, polypharmacy emerges as a critical amplifier of immune frailty in the elderly, acting through intertwined mechanisms of microbiota disruption and direct immunomodulation.

### 9.3. Multimorbidity and Cumulative Infection Risk

Multimorbidity, a condition defined by the occurrence of two or more medical conditions, is no longer the exception but the rule in hospitalized older adults, and its implications for infection risk extend far beyond the simple addition of individual comorbidities [64]. Multimorbidity is associated per se with an increased risk of hospitalizations [65], and the coexistence of chronic conditions such as diabetes, chronic kidney disease, COPD, and heart failure generates a network of converging pathophysiological alterations such as persistent low-grade inflammation, impaired mucociliary clearance, endothelial dysfunction, metabolic dysregulation, and reduced leukocyte competence [66]. These factors do not act in isolation but reinforce each other, producing a state of “immune frailty” that markedly increases vulnerability to both community-acquired and nosocomial infections [66].

Importantly, the relationship between multimorbidity and infection is not linear but cumulative: each added chronic condition compounds the risk, amplifying susceptibility to pneumonia, urinary tract infections, sepsis, and *Clostridioides difficile* infection. In a retrospective study using the Cumulative Illness Rating Scale (CIRS), older inpatients with the highest multimorbidity burden had a more than fivefold increased risk of hospital-acquired *C. difficile* infection compared with those with lower scores [67]. Similarly, in a large multicenter cohort of older adults hospitalized with Omicron variant from SARS-CoV-2 infection, multimorbidity combined with functional decline was strongly predictive of progression to severe disease (OR ≈10) [68].

Taken together, these findings underscore that multimorbidity should not be regarded merely as background noise, but as a central determinant of infection trajectories in the elderly. Its synergistic impact with malnutrition, sarcopenia, and polypharmacy generates a self-perpetuating cycle of dysbiosis, inflammation, and immune decline, amplifying infection risk and adverse outcomes.

## 10. Emerging Therapeutic and Preventive Strategies

### 10.1. Nutritional Interventions and Immunonutrition

Nutritional management is fundamental in older inpatients, where malnutrition and sarcopenia are frequent and contribute to impaired host defenses. Systematic screening with validated tools such as the Mini Nutritional Assessment (MNA), Malnutrition Universal Screening Tool (MUST), or Controlling Nutritional Status (CONUT) score allows early identification of at-risk individuals, who should promptly receive tailored nutritional support [69,70,71].

MNA is specifically developed and validated for older adults, and combines anthropometric measures, dietary assessment, and global/subjective evaluation; the short form (MNA-SF) is widely used in hospitals and long-term care [69]. MUST is instead a practical five-step instrument that evaluates BMI, recent unintentional weight loss, and the anticipated impact of acute illness on dietary intake. It is easy to implement across a wide range of clinical settings and is widely recommended for routine nutritional risk screening in hospitalized patients [70]. Finally CONUT is a laboratory-based screening tool calculated from serum albumin, total lymphocyte count, and cholesterol, reflecting both nutritional and immune status, with strong prognostic value in hospitalized patients [71].

Core nutritional interventions generally include ensuring adequate caloric intake, providing high-quality proteins to help preserve lean body mass, and addressing deficiencies of key micronutrients such as vitamin D, zinc, and selenium. These nutrients are recognized as important cofactors in several immune processes and may contribute to maintaining host defense, although the evidence in older hospitalized populations remains limited [72,73]. However, in routine internal medicine wards, nutritional assessment is often underutilized and patients frequently receive standardized diets without individualized adjustments. This approach, while practical, may overlook specific deficits and, particularly during prolonged hospitalizations, can lead to significant caloric and protein inadequacies, thereby aggravating pre-existing malnutrition and further compromising immune resilience [74,75]

### 10.2. Microbiota-Targeted Therapies (Probiotics, Prebiotics, Postbiotics, FMT)

Among the available interventions, probiotics have been the most extensively studied. Administered as live microorganisms in adequate amounts, they have shown some promise in reducing infectious complications in frail older inpatients. For example, in a recent trial involving tube-fed elderly patients treated with antibiotics, probiotic supplementation reduced the frequency of diarrhea and improved overall tolerance [76]. Prebiotics, on the other hand, act by selectively stimulating beneficial microbial taxa such as SCFA-producing bacteria, thereby enhancing anti-inflammatory pathways; however, clinical evidence in hospitalized geriatric populations is still limited and largely exploratory [77]. A further step is represented by postbiotics, consisting of inactivated microorganisms or their structural components. These compounds appear particularly attractive in frail or immunocompromised patients, as they retain immunomodulatory properties without the risks associated with live bacteria [78].

In a systematic review, postbiotics derived from heat-inactivated Lactobacillaceae and Bifidobacteriaceae strains demonstrated anti-inflammatory properties through modulation of cytokine expression, reinforcement of epithelial barrier integrity, and regulation of immune cell signaling pathways. They were also shown to rebalance Th1/Th2 and Treg/Th17 responses, indicating potential applications in inflammatory bowel disease, autoimmune disorders, and metabolic syndrome. Nevertheless, considerable study heterogeneity and risk of bias warrant cautious interpretation of these findings [79].

The most established microbiota-directed strategy is fecal microbiota transplantation (FMT), which is considered an effective option for recurrent *Clostridioides difficile* infection, including in older adults, particularly when conventional pharmacological therapies fail. While FMT has shown sustained reductions in recurrence rates and, in selected studies, decreased MDRO carriage through restoration of colonization resistance, it remains a semi-invasive intervention that is not without logistical and safety considerations. Its use should therefore be viewed as complementary rather than a first-line approach, and further research is needed to better define its long-term efficacy and optimal patient selection [80].

Taken together, probiotics, prebiotics, postbiotics, and FMT illustrate different but complementary approaches to modulating the gut microbiota in elderly inpatients. While the evidence base is steadily growing, particularly for FMT, important gaps remain regarding strain specificity, dosing, long-term safety, and applicability in immunocompromised hosts. Well-designed trials targeting frail hospitalized populations are therefore essential to define the true role of microbiota-targeted therapies in infection prevention.

### 10.3. Epigenetic Modulators and Senolytics

Epigenetic dysregulation is a recognized hallmark of aging and contributes to immune decline through altered gene expression, reduced lymphocyte diversity, and persistent low-grade inflammation. As mentioned above, pharmacological epigenetic modulators, including HDAC and DNMT inhibitors, have been explored in preclinical models for their ability to restore chromatin accessibility and partially reverse age-related immune dysfunctions [81].

Same efforts have focused on senolytics, compounds that selectively eliminate senescent cells, which accumulate with age and secrete pro-inflammatory mediators collectively known as SASP (see above). By reducing the SASP burden, senolytics such as dasatinib and quercetin have demonstrated in animal models the capacity to improve immune function, enhance vaccine responses, and lower infection susceptibility. Early human studies suggest acceptable safety profiles and possible functional benefits, but data on infection outcomes are still preliminary [82].

Although senolytics and epigenetic modulators demonstrate encouraging preclinical effects on immune rejuvenation and infection control, robust clinical data are still sparse. Translational challenges include defining appropriate patient selection, ensuring safety in frail multimorbid populations, and validating long-term efficacy through randomized controlled trials.

**Figure 1:**
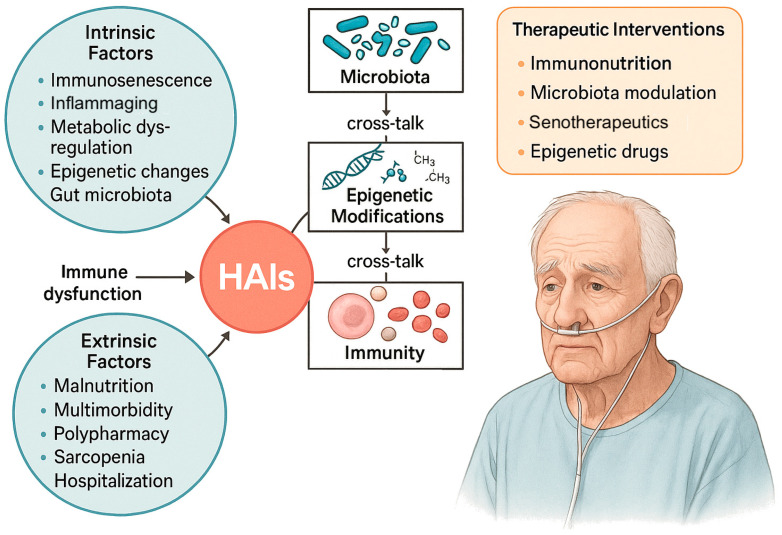
Intrinsic and extrinsic factors contributing to HAIs in older adults. Aging-related mechanisms including immunosenescence, inflammaging, metabolic dysregulation, epigenetic alterations, and gut microbiota changes, interact with clinical determinants such as malnutrition, multimorbidity, polypharmacy, sarcopenia, and hospitalization, converging to cause immune dysfunction and increased infection risk. Cross-talk between microbiota, epigenetic modifications, and immunity further amplifies vulnerability. Potential therapeutic interventions include immunonutrition, microbiota modulation (e.g., probiotics, prebiotics, FMT), senotherapeutics, and epigenetic drugs. HAIs, healthcare-associated infections.

Figure 1 shows how multiple biological mechanisms, from immunosenescence and inflammaging to metabolic and epigenetic dysregulation, microbiota shifts, and clinical amplifiers, converge to weaken host defenses in older adults, ultimately heightening their vulnerability to HAIs.

## 11. Conclusions and Future Perspectives

HAIs in older adults represent a multifactorial challenge at the crossroads between biological aging and healthcare exposure. Rather than being the consequence of a single determinant, they arise from the convergence of several processes such as immunosenescence, inflammaging, metabolic rewiring, epigenetic drift, and gut microbiota dysbiosis. These intrinsic age-related mechanisms progressively weaken host defenses, impair repair processes, and reduce the ability to clear pathogens efficiently. At the same time, extrinsic factors typical of geriatric patients including malnutrition, sarcopenia, multimorbidity, polypharmacy, and prolonged hospitalization, act as amplifiers that further increase vulnerability. Together, these elements contribute to immune frailty, persistent colonization, and a higher burden of severe infections caused by MDR organisms, ultimately worsening prognosis and clinical outcomes.

Looking ahead, the convergence of molecular research and clinical innovation offers a unique opportunity to move from reactive treatment to proactive prevention. In the short term, the most realistic interventions include systematic nutritional screening, tailored immunonutrition, and consolidated infection prevention strategies, which can already be implemented in routine clinical care. By contrast, approaches such as senolytics, epigenetic modulators, and advanced microbiota-directed therapies remain largely experimental, requiring further validation in well-designed clinical trials. From an applied perspective, feasibility and cost-effectiveness also need to be considered. While nutritional screening and infection prevention measures are low-cost, readily implementable, and supported by strong evidence, advanced approaches such as senolytics, epigenetic modulators, or fecal microbiota transplantation still face important barriers. These include safety concerns in frail multimorbid patients, logistical constraints in hospital settings, and uncertainties regarding long-term efficacy and resource allocation. Addressing these challenges will be essential to move promising concepts from bench to bedside.

In parallel, advancing the standardization of immunosenescence biomarkers and conducting multicenter longitudinal studies will be critical to support this translational shift. Developing reliable biomarkers of immune aging will be crucial to identify high-risk patients early and to tailor interventions accordingly. In this context, the application of artificial intelligence and machine learning to large multi-omics and clinical datasets may help predict individual risk, identify new therapeutic targets, and support personalized decision-making in the management of HAIs. Beyond risk stratification, these technologies could enable the construction of dynamic “immune-aging profiles” that integrate molecular, microbial, and clinical dimensions, paving the way for precision prevention and truly individualized therapeutic approaches. Such advances hold the promise not only of reducing the burden of infections in frail older adults but also of reshaping the broader paradigm of healthy aging.

## Figures and Tables

**Table 1:** Molecular Mechanisms of Immunosenescence and Impact on HAI.

Domain	Molecular Mechanisms	Functional Consequences	Impact on HAIs	Potential Interventions
**Innate Immunity**	↓ TLR7/9 signaling, ↓ IFN-α & IL-12; ↓ MHC-II, CD80/86; impaired neutrophil chemotaxis & phagocytosis; ↓ NK cytotoxicity; dysregulated complement activation (↑ C3a, C5a)	Defective pathogen recognition; impaired antigen presentation; reduced clearance of bacteria/viruses; excessive bystander inflammation	Delayed infection control; persistence of pathogens; increased tissue damage with invasive devices	TLR agonists; cytokine modulation; trained immunity
**Adaptive Immunity**	Thymic involution (↓ naïve T cells, ↓ TCR diversity); CD4+ decline, ↑ CD8+, CD28 loss; ↓ Tfh-B cell cooperation; impaired germinal center reactions	Reduced antibody affinity and memory; T-cell exhaustion; imbalance Th1/Th2/Th17; dysfunctional Treg	Lower vaccine efficacy; poor response to novel antigens; recurrent/severe HAIs	Adjuvanted vaccines; immune checkpoint modulation; T cell rejuvenation
**Inflammaging**	Chronic NF-κB activation; ↑ IL-6, TNF-α, IL-1β; accumulation of senescent cells with SASP; mitochondrial dysfunction & ↑ ROS; NLRP3 inflammasome activation	Persistent low-grade inflammation; impaired tissue repair; immunopathology	Increased baseline inflammation; higher risk of sepsis; delayed recovery from infections	Anti-IL-6 agents; senolytics; NF-κB inhibitors
**Immunometabolism**	↑ mTOR, ↓ AMPK; defective autophagy/mitophagy; altered T-cell glycolysis; impaired B-cell bioenergetics	Accumulation of damaged mitochondria; oxidative stress; defective clonal expansion; impaired antibody production	Reduced clearance of intracellular pathogens; recurrent infections; autoimmunity	mTOR inhibitors (rapalogs); AMPK activators; autophagy inducers
**Epigenetic Regulation**	DNA methylation drift (global hypomethylation, site-specific hypermethylation); histone acetylation/methylation changes; altered miRNA/lncRNA profiles	Dysregulated gene expression (immune signaling, PRRs, cytokines); persistent NF-κB activation; impaired antimicrobial pathways	Increased susceptibility to colonization and nosocomial infections	HDAC inhibitors; DNMT inhibitors; epigenetic rejuvenation strategies
**Gut Microbiota**	↓ Diversity (α-diversity); ↓ SCFAs (butyrate, acetate, propionate); loss of Faecalibacterium and Roseburia; ↑ opportunistic Enterobacterales; “leaky gut” (↑ LPS translocation)	Reduced Treg activity; impaired mucosal tolerance; chronic inflammation; endotoxemia	Colonization by *C. difficile*, ESBL-producing Enterobacteriaceae, carbapenem-resistant *K. pneumoniae*, VRE	Probiotics; prebiotics; postbiotics; FMT

The table illustrates the principal domains of immune aging including innate and adaptive immunity, inflammaging, immunometabolism, epigenetic regulation, and gut microbiota, together with their underlying molecular mechanisms, functional consequences, and clinical impact on HAI susceptibility and severity in the elderly population. Abbreviations: ↓, decrease; ↑, increase; HAI, healthcare-associated infection; TLR, Toll-like receptor; MHC, major histocompatibility complex; NK, natural killer; SASP, senescence-associated secretory phenotype; ROS, reactive oxygen species; mTOR, mechanistic target of rapamycin; AMPK, AMP-activated protein kinase; LPS = lipopolysaccharide; SCFA, short-chain fatty acid; PRRs = pattern recognition receptors; NF-κB, Nuclear Factor kappa-light-chain-enhancer of activated B cells; ESBL, extended-spectrum β-lactamase; VRE, vancomycin-resistant Enterococcus; CRE, carbapenem-resistant Enterobacteriaceae; IL, interleukin; HDAC, histone deacetylases; DNMT, DNA methyltransferases; FMT, fecal microbiota transplantation.

## Data Availability

No new data were created or analyzed in this study. Data sharing is not applicable.
